# Income differences in time to colon cancer diagnosis

**DOI:** 10.1002/cam4.6999

**Published:** 2024-08-03

**Authors:** Laura E. Davis, Erin C. Strumpf, Sunil V. Patel, Alyson L. Mahar

**Affiliations:** ^1^ Department of Epidemiology, Biostatistics and Occupational Health McGill University Montreal Canada; ^2^ ICES Toronto Canada; ^3^ Department of Economics McGill University Montreal Canada; ^4^ Department of Surgery Queen's University Kingston Canada; ^5^ School of Nursing Queen's University Kingston Canada

**Keywords:** colon cancer, diagnostic delay, diagnostic interval, diagnostic pathways, income, inequalities, inequities, neoplasms

## Abstract

**Introduction:**

People with low income have worse outcomes throughout the cancer care continuum; however, little is known about income and the diagnostic interval. We described diagnostic pathways by neighborhood income and investigated the association between income and the diagnostic interval.

**Methods:**

This was a retrospective cohort study of colon cancer patients diagnosed 2007–2019 in Ontario using routinely collected data. The diagnostic interval was defined as the number of days from the first colon cancer encounter to diagnosis. Asymptomatic pathways were defined as first encounter with a colonoscopy or guaiac fecal occult blood test not occurring in the emergency department and were examined separately from symptomatic pathways. Quantile regression was used to determine the association between neighborhood income quintile and the conditional 50th and 90th percentile diagnostic interval controlling for age, sex, rural residence, and year of diagnosis.

**Results:**

A total of 64,303 colon cancer patients were included. Patients residing in the lowest income neighborhoods were more likely to be diagnosed through symptomatic pathways and in the emergency department. Living in low‐income neighborhoods was associated with longer 50th and 90th‐percentile symptomatic diagnostic intervals compared to patients living in the highest income neighborhoods. For example, the 90th percentile diagnostic interval was 15 days (95% CI 6–23) longer in patients living in the lowest income neighborhoods compared to the highest.

**Conclusion:**

These findings reveal income inequities during the diagnostic phase of colon cancer. Future work should determine pathways to reducing inequalities along the diagnostic interval and evaluate screening and diagnostic assessment programs from an equity perspective.

## 1 INTRODUCTION

Early detection of colon cancer through organized screening programs and efficient diagnostic pathways is critical for improving overall survival.^1^, ^2^ Studies have shown that prolonged diagnostic intervals can result in adverse outcomes, including later stage at diagnosis, increased patient anxiety, and worse survival rates.^3^, ^4^, ^5^ Defined as the time from screening or initial presentation of symptoms to the cancer diagnosis, the diagnostic interval is a modifiable factor that can improve adverse outcomes.^6^, ^7^

Barriers to navigating the healthcare system for a cancer diagnosis exist at the patient, provider and health system levels.^8^ These barriers are prevalent for all patients, but they disproportionately effect individuals facing structural inequities, such as poverty, leading to inequalities in cancer outcomes.^9^ Cancer patients experiencing low income are more likely to have worse stage at diagnosis, and lower rates of survival and screening, however, little is known about income inequalities in the diagnostic interval specifically.^10^, ^11^, ^12^, ^13^, ^14^, ^15^, ^16^ To our knowledge, only one other study has examined the colon cancer diagnostic interval by income, reporting a median diagnostic interval of 6.5 days longer in patients living in low‐income neighborhoods compared to high‐income neighborhoods, however, the objective of this study was to examine multiple factors associated with the diagnostic interval and not income specifically.^16^

Thus, the objective of this work was to describe diagnostic interval characteristics by neighborhood income quintile and estimate the associations between neighborhood income and the length of diagnostic interval. By conducting this research, we seek to contribute to the understanding of income inequalities during the diagnostic interval and subsequently cancer outcomes. The findings of this study will provide valuable insights for developing and evaluating targeted interventions to reduce inequities in diagnostic care and improve patient outcomes.

## 2 METHODS

### 2.1 Study design

This was a population‐based retrospective cohort study using linked routinely collected administrative healthcare databases (i.e., data collected as part of routine healthcare process) held at ICES (formerly the Institute for Clinical Evaluative Sciences) in Ontario, Canada. Ethics approval was obtained from McGill University Research Ethics Board (#A04‐M37‐22A), and we followed privacy guidelines set out by ICES. Written informed consent was waived by ICES and the Research Ethics Board.

### 2.2 Data sources

Data were obtained from data holdings at ICES which houses data on all publicly funded healthcare interactions in Ontario, including cancer diagnostic procedures and investigations. ICES is an independent, non‐profit research institute whose legal status under Ontario's health information privacy law allows it to collect and analyze health care and demographic data without consent, for health system evaluation and improvement. Datasets were linked using unique encoded identifiers and analyzed at ICES. Datasets are described in detail in Table S1. Briefly, we used the Ontario Cancer Registry (OCR),^17^ hospitalization data from the Canadian Institute of Health Information (CIHI) Same Day Surgery and Discharge Abstract Database (DAD),^18^ emergency department data from the National Ambulatory Care Reporting System,^19^ physician claims data from Ontario Health Insurance Plan (OHIP) billing dataset and demographic data from the Registered Persons Database (RPDB).

### 2.3 Study population

All residents of Ontario have universal, publicly funded health insurance, including primary and cancer care coverage, through a government‐administered single‐payer system. The study included Ontarian adults with a first colon cancer diagnosis (International Classification of Diseases for Oncology (ICD‐O‐3) codes C18.0, C18.2–C18.9) registered between January 1, 2007, and December 31, 2019, in the OCR. We excluded individuals who had a death date before their diagnosis date, those diagnosed with multiple cancers on the same day, no OHIP eligibility 2 years before the diagnosis, those for whom the first contact encounter for the diagnostic interval could not be identified and who had missing information on income.

### 2.4 Measures

#### 2.4.1 Exposure

Measuring the diagnostic interval must occur in provincial ICES datasets where physician billing information is linked to hospital records and the cancer registry. Currently, these data are not linked to individual measures of income. As a result, we used neighborhood income as a proxy for individual income while understanding the limitations of this approach.^20^ In the absence of individual data, neighborhood measures are commonly used to approximate individual income in cancer studies.^21^, ^22^ Neighborhood income quintiles were obtained from the RPDB and measured using the Postal Code Conversion File (PCCF+) linked to the postal code at diagnosis. The PCCF+ neighborhood income variable is created by Statistics Canada using census summary data and represents the median, before‐tax, household‐adjusted income within each dissemination area.^23^ Dissemination areas are Statistics Canada's smallest geographical unit representing approximately 400–700 individuals per area.^24^ Quintiles are created by ranking dissemination areas within each census metropolitan area, census agglomeration or other region from lowest to highest, then dividing into fifths. Individuals in quintile 1 reside in neighborhoods with the lowest income and individuals in quintile 5 reside in neighborhoods with the highest income.

#### 2.4.2 Outcome

Following the Aarhus statement, we defined the diagnostic interval as the number of days from the earliest healthcare encounter (physician visit or hospital admission) related to colon cancer to the diagnosis date, usually the first malignant biopsy date.^6^ We modified established methods from Groome et al. and Webber et al.^25^, ^26^ to define the earliest healthcare encounter using different lookback periods for each encounter category. These methods are described in detail elsewhere and have been used in CRC and breast cancer.^25^, ^27^, ^28^ Briefly, we identified and categorized encounters occurring more frequently in the 0–3 months compared to the 24–27 months before diagnosis and determined cancer‐related lookback periods for each encounter category using statistical process control.^29^ We identified referring physician visits for all procedure‐based encounters as the first visit with that referring physician that occurred less than 365 days from the procedure date. The earliest encounter was defined as the first eligible healthcare encounter, and we calculated the diagnostic interval as the number of days between the first encounter date or referring physician date to the diagnosis date. Our modification included extending the lookback period to 2 years to identify encounters, including all encounters that demonstrate an increase in the 0–3 months before diagnosis regardless of relation to colon cancer, and using a more liberal cut‐off for the statistical process control.

#### 2.4.3 Diagnostic interval characteristics

Other diagnostic interval variables are described in detail in Table S2. Variables describing the diagnostic interval were measured along the diagnostic interval and included: first encounter type, symptomatic or asymptomatic pathway, referring physician as first contact, receipt of lower gastrointestinal (GI) endoscopies, number of visit days, and the summary of the diagnostic pathway. The first encounter type was defined as the earliest category of encounter that occurred on the first encounter date; patients could have more than one encounter on their first encounter date. An asymptomatic pathway was defined as an interval where the first encounter was a guaiac fecal occult blood test (gFOBT) or lower GI endoscopy that occurred alone or in combination with a consultation and did not occur in the emergency department (ED). An interval was considered symptomatic if there was a symptom‐related encounter or nonscreening procedure as the first encounter or if the first encounter occurred in the ED. The diagnostic pathway was summarized in 9 possible pathways: (1) asymptomatic; (2) lower GI endoscopy alone, presenting in the ED; (3) lower GI endoscopy alone, not presenting in the ED; (4) lower GI endoscopy and imaging presenting in the ED; (5) lower GI endoscopy and imaging not presenting in the ED; (6) imaging alone, presenting in the ED; (7) imaging alone, not presenting in the ED; (8) no lower GI endoscopy or imaging presenting in the ED; (9) no lower GI endoscopy or imaging not presenting in the ED.^30^

#### 2.4.4 Patient characteristics

Covariates are detailed in Table S2. Demographic and cancer‐related variables were measured in the year of diagnosis. Comorbidities were measured using the Elixhauser comorbidity index, which measured hospitalizations 2 years before cancer diagnosis and was dichotomized as ≥4 and <4.^31^ Rural residence was measured by linking postal codes at the time of diagnosis to the Rurality Index of Ontario (RIO), a function of population size, distance to family practitioners and travel time to access healthcare.^32^ RIO values were dichotomized as ≥45 for rural and <45 for urban residences.^32^ Histology and TNM stage at diagnosis were obtained from the OCR. Stage at diagnosis represents the best International Union for Cancer Control and American Joint Committee on Cancer stage, a combination of the Collaborative Staging approach and data from medical records at regional cancer centres. Stage was broadly categorized as stages I/II/III/IV and unknown. Histology was dichotomized as adenocarcinoma and nonadenocarcinoma using morphology codes.

### 2.5 Statistical analysis

We described the cohort demographics and disease characteristics by neighborhood income quintile. The diagnostic interval in days and its characteristics were described by symptom status and neighborhood income quintile. Means, medians and interquartile ranges were presented for continuous variables and numbers and proportions for categorical variables. Chi‐squared tests were used to test significant differences between categorical variables and neighborhood income quintiles.

Quantile regression was used to estimate the association between neighborhood income quintile and the conditional median diagnostic interval, stratified by symptomatic and asymptomatic pathways.^33^ Quantile regression is useful in situations where the outcome is left‐skewed, such as with the diagnostic interval and allows us to examine inequalities at each percentile.^33^ We present effect estimates at the 50th and 90th percentile to understand the association of neighborhood income at the median diagnostic interval and the 90th percentile diagnostic interval where patients have the longest intervals and are most at risk for poor outcomes. Estimates at the 10th percentile were initially explored to understand the association of income for the patients with the shortest diagnostic intervals but results were null due to a lack of variation in the diagnostic interval at this percentile between income groups (Table S3). We present 95% confidence intervals (CI) and *p*‐values <0.05 represent statistical significance. Multivariable models included continuous age, sex, rural residence, and year of diagnosis. Comorbidities and stage at diagnosis were conceptualized as being on the causal pathway and, therefore, not included in the multivariable models.^34^ We performed an additional analysis stratifying models by stage at diagnosis to determine any differences in the association between neighborhood income and the diagnostic interval at different stages. SAS version 9.4 was used for all analyses.

## 3 RESULTS

### 3.1 Study cohort

**FIGURE 1:**
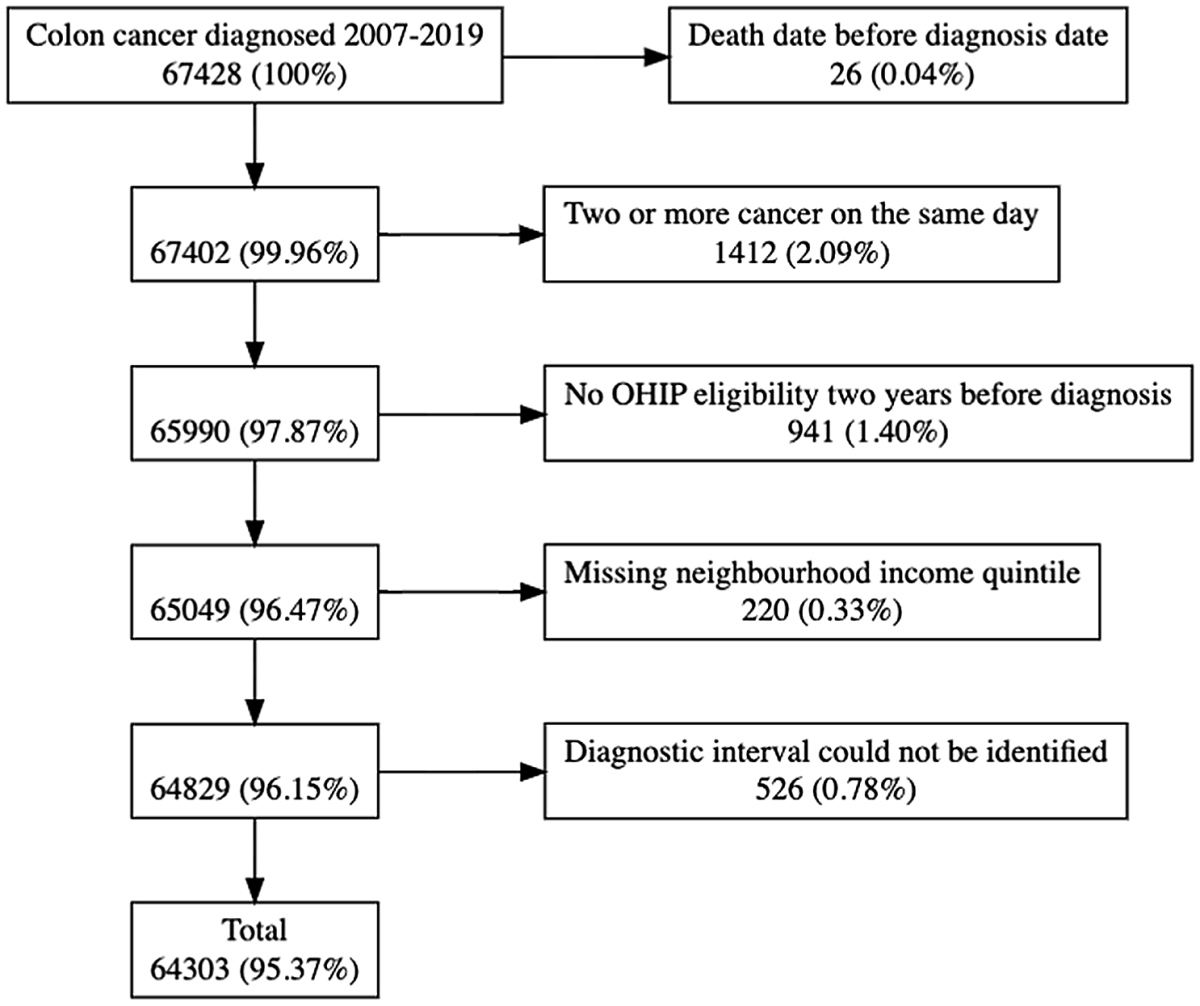
Cohort exclusions.

A total of 67,428 individuals were diagnosed with colon cancer between 2007 and 2019. Of 64,303 patients 3126 were excluded for a final cohort (Figure 1).

There were some demographic and cancer differences by neighborhood income quintile (Table 1). The median age at diagnosis ranged from 73 (IQR 63–81) for individuals living in neighborhoods with the lowest income and 71 (IQR 62–81) for individuals in the highest neighborhood income quintile. Individuals residing in the lowest income neighborhoods were more likely to be female, have more comorbidities, live in rural areas, have missing stage and less likely to be diagnosed at stage 1 compared to individuals living in the highest income neighborhoods.

**TABLE 1:** Demographic and disease characteristics by neighborhood income quintile (*N*, column percent %).

Variables	Total (*N* = 64,303)	Quintile 1 (lowest income) (*N* = 13,060)	Quintile 2 (*N* = 13,502)	Quintile 3 (*N* = 12,808)	Quintile 4 (*N* = 12,437)	Quintile 5 (highest income) (*N* = 12,496)	*p*‐value*
Age at diagnosis							
≤50	4394 (6.8)	840 (6.4)	837 (6.2)	869 (6.8)	932 (7.5)	916 (7.3)	<0.001
51–60	9018 (14.0)	1767 (13.5)	1714 (12.7)	1849 (14.4)	1816 (14.6)	1872 (15.0)	
61–70	15,436 (24.0)	2963 (22.7)	3195 (23.7)	3095 (24.2)	3072 (24.7)	3111 (24.9)	
71–80	18,746 (29.2)	3867 (29.6)	4061 (30.1)	3738 (29.2)	3615 (29.1)	3465 (27.7)	
>80	16,709 (26.0)	3623 (27.7)	3695 (27.4)	3257 (25.4)	3002 (24.1)	3132 (25.1)	
Sex							
Female	31,370 (48.8)	6671 (51.1)	6659 (49.3)	6218 (48.5)	5853 (47.1)	5969 (47.8)	<0.001
Male	32,933 (51.2)	6389 (48.9)	6843 (50.7)	6590 (51.5)	6584 (52.9)	6527 (52.2)	
Rural residence							
RIO <45	59,632 (92.7)	11,903 (91.1)	12,421 (92.0)	11,881 (92.8)	11,679 (93.9)	11,748 (94.0)	<0.001
RIO ≥45	4671 (7.3)	1157 (8.9)	1081 (8.0)	927 (7.2)	758 (6.1)	748 (6.0)	
Elixhauser comorbidities							
<4	55,435 (86.2)	10,913 (83.6)	11,511 (85.3)	11,012 (86.0)	10,960 (88.1)	11,039 (88.3)	<0.001
≥4	8868 (13.8)	2147 (16.4)	1991 (14.7)	1796 (14.0)	1477 (11.9)	1457 (11.7)	
Histology							
Other	1858 (2.9)	348 (2.7)	382 (2.8)	375 (2.9)	357 (2.9)	396 (3.2)	0.192
Adenocarcinoma	62,445 (97.1)	12,712 (97.3)	13,120 (97.2)	12,433 (97.1)	12,080 (97.1)	12,100 (96.8)	
Stage at diagnosis							
I	12,126 (18.9)	2268 (17.4)	2517 (18.6)	2460 (19.2)	2416 (19.4)	2465 (19.7)	<0.001
II	16,062 (25.0)	3309 (25.3)	3404 (25.2)	3158 (24.7)	3064 (24.6)	3127 (25.0)	
III	15,513 (24.1)	3194 (24.5)	3230 (23.9)	3077 (24.0)	3020 (24.3)	2992 (23.9)	
IV	11,193 (17.4)	2290 (17.5)	2298 (17.0)	2232 (17.4)	2198 (17.7)	2175 (17.4)	
Unknown/missing	9409 (14.6)	1999 (15.3)	2053 (15.2)	1881 (14.7)	1739 (14.0)	1737 (13.9)	

^*^ Differences between income quintiles tested using chi‐squared test.

### 3.2 Diagnostic pathway description

Features of the diagnostic pathways are described in Table 2. There were 11,378 (17.7%) patients with an asymptomatic interval. Patients living in the lowest income quintile neighborhoods were less likely to experience an asymptomatic pathway (17.4% vs. 20.4% in the highest income quintile). The first encounter for asymptomatic pathways differed slightly by neighborhood income quintile. Patients living in the lowest income quintile neighborhoods were less likely to have a lower GI scope as their first encounter (22.6% in Q1 vs. 26.2% in Q5, *p* = 0.0028) and more likely to have a gFOBT (77.4% in Q1 vs. 73.9% in Q5, *p* = 0.0033) compared to individuals residing the highest income neighborhoods.

**TABLE 2:** Features of the diagnostic interval by symptom status and income quintile (*N*, column percent %).

Asymptomatic							Symptomatic					
	Quintile 1 (lowest) (N = 1985)	Quintile 2 (*N* = 2386)	Quintile 3 (*N* = 2377)	Quintile 4 (*N* = 2309)	Quintile 5 (highest) (*N* = 2321)	*p*‐value	Quintile 1 (lowest) (*N* = 11,075)	Quintile 2 (*N* = 11,116)	Quintile 3 (*N* = 10,431)	Quintile 4 (*N* = 10,128)	Quintile 5 (highest) (*N* = 10,175)	*p*‐value
Diagnosed on index encounter date (diagnostic interval = 1 day)												
No	1890 (95.2)	2269 (95.1)	2278 (95.8)	2178 (94.3)	2187 (94.2)	0.0702	10,141 (91.6)	10,254 (92.2)	9634 (92.4)	9335 (92.2)	9320 (91.6)	0.0851
Yes	95 (4.8)	117 (4.9)	99 (4.2)	131 (5.7)	134 (5.8)		934 (8.4)	862 (7.8)	797 (7.6)	793 (7.8)	855 (8.4)	
Referring physician as first contact												
No	1756 (88.5)	2075 (87.0)	2083 (87.6)	1994 (86.4)	1994 (85.9)	0.096	9538 (86.1)	9501 (85.5)	8884 (85.2)	8548 (84.4)	8520 (83.7)	<0.0001
Yes	229 (11.5)	311 (13.0)	294 (12.4)	315 (13.6)	327 (14.1)		1537 (13.9)	1615 (14.5)	1547 (14.8)	1580 (15.6)	1655 (16.3)	
ED at index												
No	1985 (100.0)	2386 (100.0)	2377 (100.0)	2309 (100.0)	2321 (100.0)	NA	7177 (64.8)	7476 (67.3)	7023 (67.3)	6926 (68.4)	7117 (69.9)	<0.0001
Yes							3898 (35.2)	3640 (32.7)	3408 (32.7)	3202 (31.6)	3058 (30.1)	
Lower GI scope												
0	324 (16.3)	405 (17.0)	395 (16.6)	362 (15.7)	367 (15.8)	0.7301	4451 (40.2)	4286 (38.6)	3923 (37.6)	3866 (38.2)	3788 (37.2)	<0.0001
1+	1661 (83.7)	1981 (83.0)	1982 (83.4)	1947 (84.3)	1954 (84.2)		6624 (59.8)	6830 (61.4)	6508 (62.4)	6262 (61.8)	6387 (62.8)	

**FIGURE 2:**
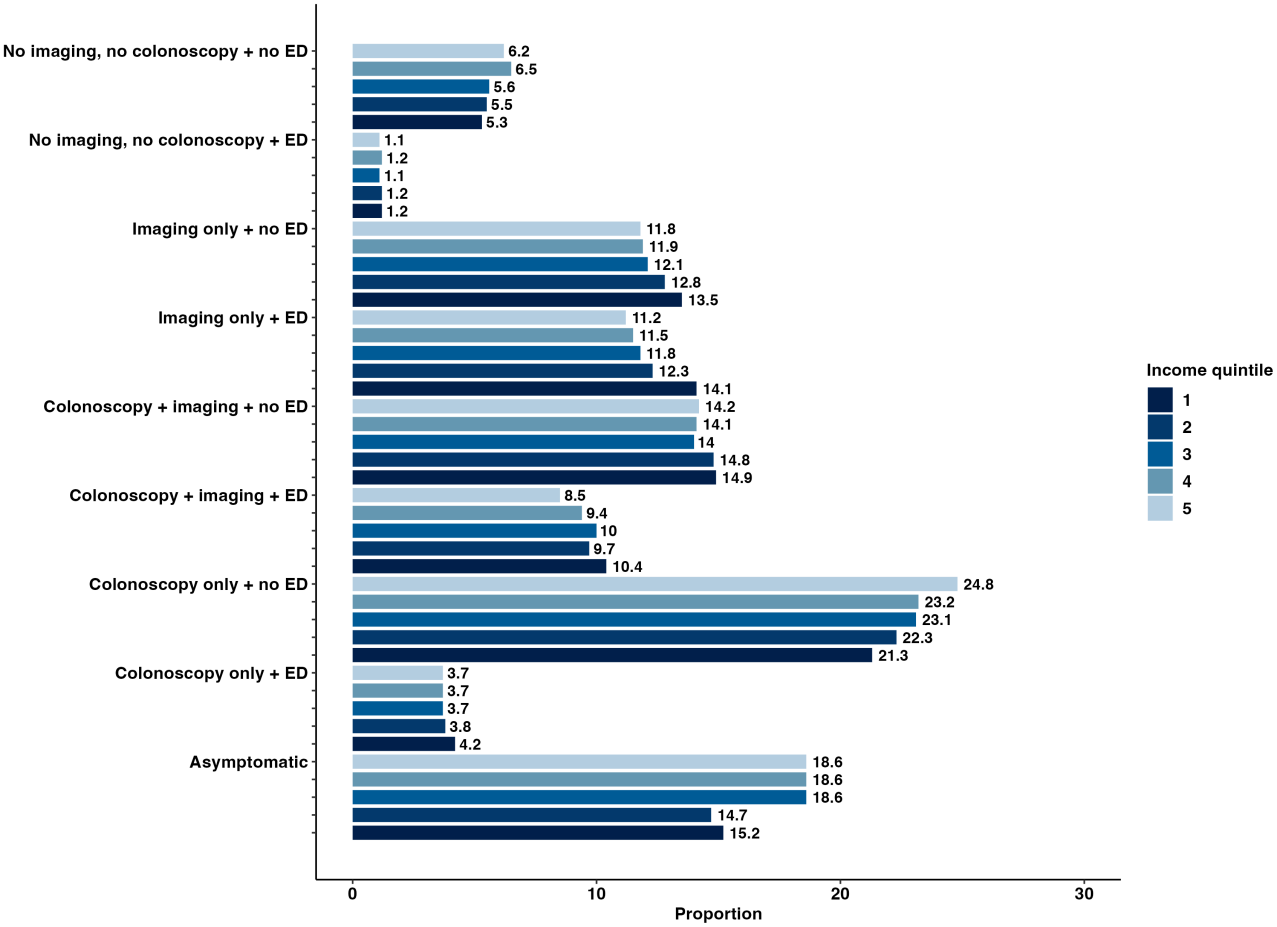
Frequency of diagnostic pathways by income quintile. (Neighborhood income quintile 1 = lowest; neighborhood income quintile 5 = highest).

82,925 (82.3%) patients had a symptomatic interval. The first encounter for symptomatic pathways was most likely to be a diagnostic code for GI signs and symptoms, followed by an emergency family physician visit. These were similar between neighborhood income quintiles (Table S4). An ED visit on the first encounter date was more likely to occur in patients living in the lowest income neighborhoods (35.2%) compared to patients living in the highest income neighborhoods (30.1%). Diagnostic pathways also differed by neighborhood income quintile, with individuals residing in the lowest income neighborhoods more likely to be diagnosed through lower GI endoscopy with imaging in the ED (12.2% vs. 10.4% in quintile 5) and less likely to be diagnosed through colonoscopy only outside the ED (25.1% vs. 30.5% in quintile 5) compared to individuals residing in the highest income neighborhoods (Figure 2).

### 3.3 Diagnostic interval description

The diagnostic interval overall was 108 days (IQR 31–243 days) with a 90th percentile of 383 days. Patients with asymptomatic pathways had shorter median and 90th percentile diagnostic intervals compared to symptomatic pathways (median 71 days [IQR 35–137, 90th percentile 230 days] vs. 148 median 121 days [IQR 29–273, 90th percentile 404 days], respectively). Asymptomatic median and 90th percentile diagnostic intervals were similar across neighborhood income quintiles in descriptive analysis, ranging from 71 days (IQR 36–130, 90th percentile 222 days) among individuals residing in the lowest income neighborhoods to 70 days (IQR 33–144, 90th percentile 228 days) in the highest income neighborhoods (Table S5). Symptomatic diagnostic intervals ranged from 126 days (IQR 31–280, 90th percentile 410 days) among individuals residing in the lowest income neighborhoods to 118 days (IQR 28–267, 90th percentile 400 days) in the highest income neighborhoods (Table 3).

**TABLE 3:** Median and 90th percentile diagnostic interval by symptom status and patient and disease characteristics (days, symptomatic pathways only).

Symptomatic										
Variable	Quintile 1 (lowest income)		Quintile 2		Quintile 3		Quintile 4		Quintile 5 (highest income)	
	Median (IQR)	90th pct	Median (IQR)	90th pct	Median (IQR)	90th pct	Median (IQR)	90th pct	Median (IQR)	90th pct
Diagnostic interval overall	126 (31–280)	410	124 (31–279)	410	122 (30–273)	406	118 (28–267)	400	116 (27–260)	392
Stage at diagnosis										
Stage I	160 (72–294)	408	153 (61–291)	407	148 (60–286)	396	153 (26–284)	405	153 (57–283)	400
Stage II	121 (30–277)	412	125 (35–270)	413	124 (35–266)	404	115 (29–261)	392	116 (30–256)	388
Stage III	126 (33–284)	405	119 (30–274)	400	116 (27–272)	407	113 (28–258)	402	113 (29–248)	383
Stage IV	85 (13–214)	382	82 (15–226)	385	79 (13–228)	388	70 (12–227)	376	59 (11–205)	360
Stage unknown/missing	145 (36–316)	435	138 (36–314)	434	139 (35–315)	434	140 (40–303)	423	137 (34–294)	428
Age at index (categorical)										
≤50	105 (18–246)	386	103 (22–229)	380	102 (20–258)	386	75 (17–207)	373	97 (22–211)	346
51–60	103 (19–233)	371	100 (22–240)	372	96 (17–214)	364	94 (19–226)	346	93 (19–206)	346
61–70	121 (29–265)	392	113 (29–248)	383	111 (29–254)	386	113 (25–255)	389	112 (27–257)	387
71–80	129 (35–286)	408	127 (35–284)	416	128 (38–282)	407	124 (31–272)	403	121 (31–260)	388
>80	147 (40–304)	447	144 (39–309)	445	147 (38–311)	442	142 (39–299)	434	141 (35–293)	425
Sex										
Female	137 (39–291)	418	135 (35–290)	422	128 (31–283)	413	126 (31–277)	408	120 (29–264)	395
Male	117 (25–266)	399	113 (29–261)	394	116 (30–263)	398	110 (26–256)	392	113 (26–254)	388
RIO at index										
Urban <45	128 (31–284)	413	125 (32–281)	411	123 (30–275)	407	119 (28–270)	400	117 (28–261)	395
Rural≥45	114 (28–228)	376	116 (28–255)	391	106 (29–249)	392	110 (26–245)	399	106 (22–235)	343
Elixhauser										
<4	119 (29–264)	398	116 (30–262)	398	114 (29–260)	396	112 (26–255)	393	110 (25–247)	382
≥4	173 (48–331)	466	171 (47–338)	467	164 (50–332)	464	168 (42–321)	451	170 (50–321)	438
Histology										
Other	113 (29–265)	416	113 (22–287)	423	112 (22–256)	399	113 (23–273)	410	111 (16–285)	433
Adenocarcinoma	127 (31–280)	409	124 (32–279)	409	122 (31–274)	407	118 (28–267)	400	116 (28–256)	390
Received at least one lower GI scope										
0	102 (10–266)	408	98 (11–257)	407	97 (11–255)	408	84 (10–238)	395	92 (8–240)	394
1+	141 (49–287)	411	136 (49–286)	411	136 (45–281)	404	136 (43–281)	402	130 (42–268)	390

**FIGURE 3:**
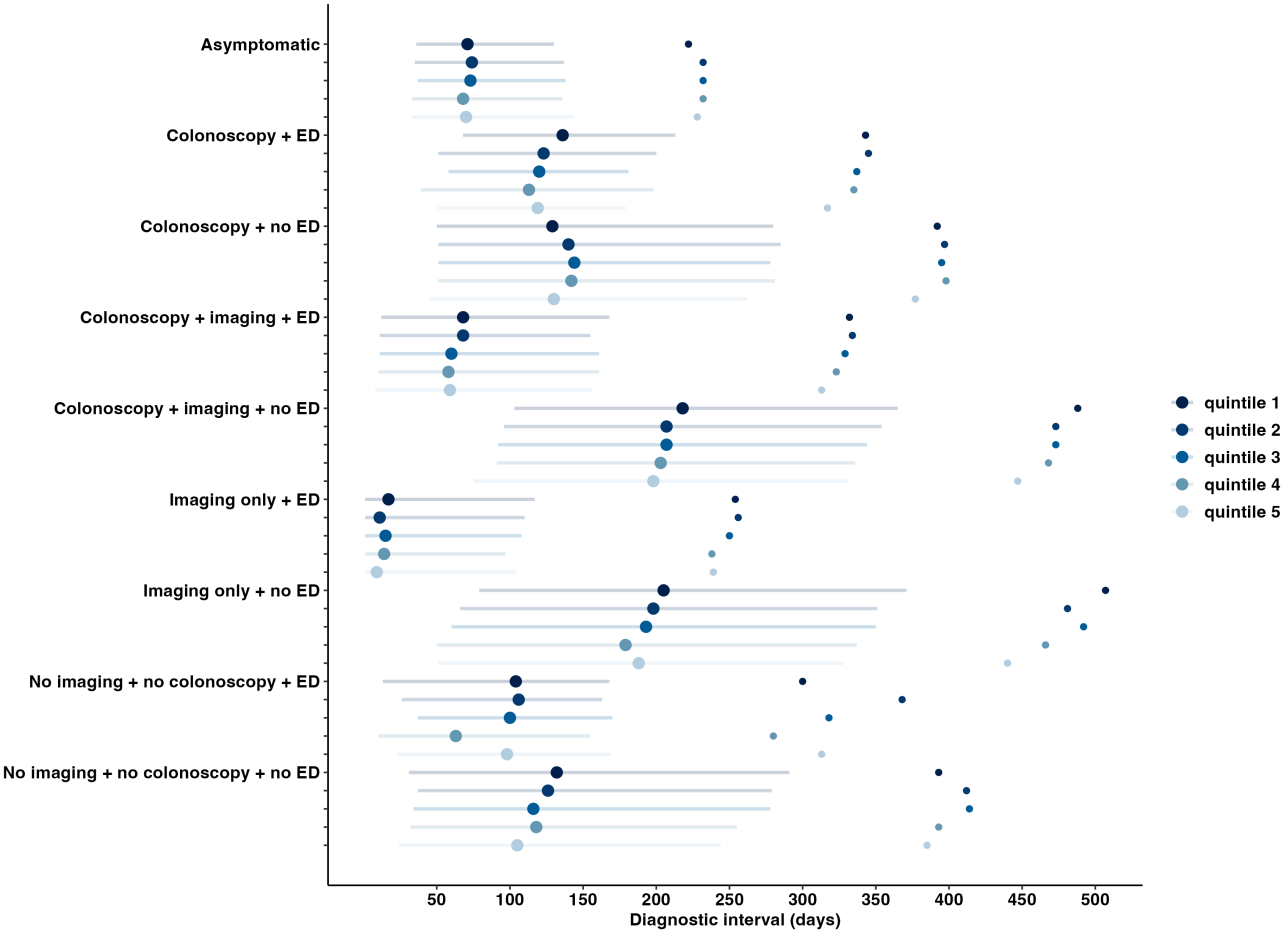
Median and interquartile range diagnostic interval for diagnostic pathway by income quintile (Dots on the far right represent the 90th percentile, dots within the lines represent the 50th percentile, with the end of the left side being the 25th percentile and the end of the right side the 75th percentile).

Older patients, women, those with comorbidities, or earlier stages had longer diagnostic intervals in both asymptomatic and symptomatic pathways. The nine diagnostic pathways had different diagnostic intervals, and these differed by income quintile, with individuals with the lowest income quintile generally experiencing longer diagnostic intervals across symptomatic pathways and similar intervals for the asymptomatic pathway compared to people with the highest income quintile (Figure 3).

### 3.4 Quantile regression models

For asymptomatic pathways, income was only significantly associated with the diagnostic interval at the 50th percentile, with patients in the three lowest income quintiles experiencing longer diagnostic intervals compared to patients in the highest income quintile. For symptomatic pathways, the three lowest income quintiles were associated with a longer 50th and 90th percentile diagnostic interval compared to patients in the highest income quintile (Table 4). For example, the 90th percentile diagnostic interval was 15 days (95% CI 6–23) longer for patients in the lowest income quintile compared to the highest. After stratifying by stage, having low income was significantly associated with the diagnostic interval for asymptomatic patients with unknown or missing stage and symptomatic patients at stages 3 and 4 (Table S6). For example, having the lowest income for symptomatic stage 3 patients was associated with a diagnostic interval that was 23 days (95% CI 8–38) longer compared to patients with the highest income.

**TABLE 4:** Quantile regression for the effect of income on the diagnostic interval stratified by symptom status (reference = Quintile 5 (highest), estimates are in days).

Model	Unadjusted				Adjusted^a^			
	50th percentile		90th percentile		50th percentile		90th percentile	
	Estimate (95% CI)	*p*‐value	Estimate (95% CI)	*p*‐value	Estimate (95% CI)	*p*‐value	Estimate (95% CI)	*p*‐value
Asymptomatic								
Intercept^b^	70.00 (65.86–74.14)	<0.0001	228.00 (216.13–239.87)	<0.0001	48.77 (39.85–57.68)	<0.0001	144.23 (130.48–157.97)	<0.0001
Quintile 1 (lowest)	1.00 (−4.38–6.38)	0.2825	−6.00 (−26.70–14.70)	0.8742	4.83 (0.27–9.39)	0.0089	−7.96 (−15.92‐(−0.01)	0.3745
Quintile 2	4.00 (−2.13–10.13)		4.00 (−15.88–23.88)		5.08 (−0.09–10.26)		−4.65 (−12.50–3.19)	
Quintile 3	3.00 (−2.43–8.43)		4.00 (−12.91–20.91)		7.22 (2.51–11.93)		−2.64 (−10.47–5.19)	
Quintile 4	−2.00 (−7.85–3.85)		4.00 (−15.25–23.25)		0.72 (−4.68–6.12)		−3.15 (−10.15–3.86)	
Symptomatic								
Intercept	116.00 (111.80–120.20)	<0.0001	392.00 (385.61–398.39)	<0.0001	108.48 (99.68–117.27)	<0.0001	410.26 (395.75–424.77)	<0.0001
Quintile 1 (lowest)	10.00 (4.42–15.58)	0.0046	18.00 (9.64–26.36)	<0.0001	10.04 (4.37–15.71)	0.0056	14.76 (6.30–23.23)	0.0051
Quintile 2	8.00 (1.63–14.38)		18.00 (9.42–26.58)		5.73 (0.40–11.06)		11.53 (3.94–19.12)	
Quintile 3	6.00 (0.52–11.48)		14.00 (5.27–22.73)		5.65 (0.12–11.19)		9.55 (2.48–16.61)	
Quintile 4	2.00 (−4.44–8.44)		8.00 (−1.02–17.02)		1.08 (−4.45–6.60)		6.80 (−0.87–14.47)	

Abbreviation: CI, confidence interval.

^a^ Adjusted models for age, sex, rural residence and diagnosis year.

^b^ Intercept interpreted as the diagnostic interval at baseline for those at median age (65 years), of male sex, living in urban areas and diagnosed in 2019).

## 4 DISCUSSION

This study found significantly longer symptomatic diagnostic intervals for patients residing in neighborhoods with the lowest income compared to those with the highest income, with increasing disparities with increasing stage at diagnosis. Our study found that the median and 90th percentile diagnostic interval for symptomatic pathways was 10 and 15 days longer for patients with the lowest income compared to those with the highest income. Smaller or no differences were found in the diagnostic interval by income for patients with asymptomatic pathways. Other studies examining inequalities in the diagnostic interval have found longer diagnostic intervals for patients residing in rural areas, women and immigrants.^35^, ^36^, ^37^ These studies demonstrated a median interval that ranged from 18 days longer for rural patients to 5 days longer for new immigrants.^35^, ^36^, ^37^ One other study demonstrated a median diagnostic interval of 6.5 days longer in patients living in low‐income neighborhoods compared to high‐income neighborhoods.^16^

Regardless of income, we found that patients with asymptomatic pathways had much shorter diagnostic intervals compared to symptomatic pathways, but patients with the lowest income were less likely to have asymptomatic diagnostic pathways and more likely to have pathways that included presenting in the ED compared to patients with the highest income. This finding reflects similar known inequities in screening rates by income, gender and immigration status.^38^ Moreover, asymptomatic pathways in our study reflect screening status as demonstrated by the similarity between the proportion of patients with asymptomatic pathways (18%) and previous studies indicating screening rates of 17% in Ontario.^30^, ^39^

### 4.1 Implications and future directions

Given the critical importance of timely diagnosis and treatment for outcomes such as patient anxiety and stage at diagnosis, the income‐based differences we found in our study contribute to significant inequities within the cancer system. Delays in diagnosis for patients experiencing low income may compound with disparities in wait times across other aspects of the cancer care continuum, such as for treatment, and result in worse overall outcomes. For instance, a meta‐analysis has shown that even a four‐week delay between surgery and adjuvant treatment for colon cancer patients could increase the risk of mortality by 9%–13%.^3^ Such delays, coupled with well‐known disparities in receiving cancer treatment, lead to substantial differences in cancer outcomes by income.^10^

The cancer system, especially in the diagnostic phase, is complex, fragmented, and often unfamiliar to patients. Well‐educated and affluent patients may be able to absorb complex medical information, advocate for themselves, and have access to formal and informal healthcare networks, which are largely inaccessible to individuals experiencing low income or other structural disadvantages.^40^ Population based screening programs, rapid assessment programs and patient navigation can improve access to cancer care and aims to reduce the time to diagnosis, especially for vulnerable populations.^41^ Future research should assess these programs' effectiveness within subgroups of underserved individuals. Additionally, to implement and improve interventions aimed at shortening the diagnostic interval and improving outcomes, research should examine the pathways through which the diagnostic interval can be reduced and how these pathways may differ among structurally disadvantaged groups. For example, continuity of care with a regular family physician could facilitate screening and increase the likelihood of reporting signs and symptoms earlier, potentially resulting in shorter diagnostic intervals for all patients, but may be especially important for patients experiencing low‐income.^42^, ^43^

### 4.2 Strengths and limitations

This study has several strengths. We used routinely collected administrative data in Ontario that captures almost all cancer patients in the province; therefore, our results reflect real‐world inequalities occurring in Ontarian colon cancer patients. We also used a modified definition of the diagnostic interval, allowing for more extended lookback periods to capture cancer and noncancer‐related diagnoses and procedures. This method might more likely capture intervals in patients experiencing diagnostic pathways that deviate from guideline recommendations. Finally, we used a conceptual model to determine the association between income and the diagnostic interval, which does not control for causal pathway variables.^34^

This study has limitations. Most importantly, individual income or other individual socioeconomic measures could not be obtained. While neighborhood income may represent a measure of the neighborhood environment, it is difficult to interpret, especially within Ontario, where neighborhoods can vary from a one‐block radius to 100 s of kilometers.^46^ In the absence of individual data, we used neighborhood income to approximate individual income.^21^, ^22^ Studies estimating misclassification of individual income using neighborhood measures have demonstrated an attenuation of the effect of income on health outcomes when using neighborhood income instead of individual income.^44^, ^45^ Therefore, we hypothesize that our results may underestimate the disparities in diagnostic interval by income.^44^ This limitation further stresses the importance of linking individual socioeconomic variables to rich, routinely collected administrative datasets. Second, we were unable to confirm screening status in the administrative data and therefore had to approximate screening with asymptomatic pathways. It is possible that individuals may have received a colonoscopy for reasons other than screening, which might overestimate the number of individuals screened in our study. However, since the screening rates in our study were similar to those in the literature, we assume this misclassification is small. Finally, while our method for creating the diagnostic interval has been outlined in detail and used previously, it has yet to be validated due to limited access to detailed linked data.^25^, ^26^

## 5 CONCLUSION

We found a meaningful differences in the diagnostic interval and pathways, with patients living in the lowest income neighborhoods less likely to be diagnosed through asymptomatic pathways, more likely to be diagnosed in the ED and having longer symptomatic diagnostic intervals compared to their high income counterparts. Future work should examine inequalities in the diagnostic interval by individual income and among other vulnerable groups and determine pathways to reducing inequalities along the diagnostic interval, such as through improved access to screening programs, diagnostic navigation programs or regular contact with a family physician.

## AUTHOR CONTRIBUTIONS

**Laura E. Davis:** Conceptualization (equal); formal analysis (equal); funding acquisition (equal); methodology (equal); visualization (equal); writing – original draft (equal); writing – review and editing (equal). **Erin C. Strumpf:** Conceptualization (equal); funding acquisition (equal); methodology (equal); supervision (equal); writing – review and editing (equal). **Sunil V. Patel:** Methodology (equal); writing – review and editing (equal). **Alyson L. Mahar:** Conceptualization (equal); funding acquisition (equal); methodology (equal); supervision (equal); writing – review and editing (equal).

## FUNDING INFORMATION

This study was supported by ICES, which is funded by an annual grant from the Ontario Ministry of Health (MOH) and the Ministry of Long‐Term Care (MLTC). This study also received funding from the Canadian Institutes of Health Research (CIHR), the Canadian Cancer Society (MEGAN‐CAN), the Canadian Centre for Applied Research in Cancer Control (ARCC) and Fonds de recherche du Québec—Santé (FRQS).

## CONFLICT OF INTEREST STATEMENT

The authors have no conflicts of interest.

## ETHICS STATEMENT

Approval of the research protocol by an Institutional Review Board: Ethics approval was obtained from McGill University Research Ethics Board (#A04‐M37‐22A), and we followed privacy guidelines set out by ICES (formerly the Institute for Clinical Evaluative Sciences). Informed Consent: The use of the data in this project is authorized under section 45 of Ontario's PErsonal Health Information Act (PHIPA) and does not require review by a Research Ethics Board. Registry and the Registry No. of the study/trial: N/A. Animal Studies: N/A.

## DISCLAIMERS

This document used data adapted from the Statistics Canada Postal Code OM Conversion File, which is based on data licensed from Canada Post Corporation, and/or data adapted from the Ontario Ministry of Health Postal Code Conversion File, which contains data copied under license from Canada Post Corporation and Statistics Canada. Parts of this material are based on data and information compiled and provided by the Canadian Institute of Health Information (CIHI). However, the analyses, conclusions, opinions, and statements expressed herein are those of the authors, and not necessarily those of the CIHI. Parts of this material are based on data and information provided by Ontario Health (OH). The opinions, results, views, and conclusions reported in this article are those of the authors and do not necessarily reflect those of OH. No endorsement by OH is intended or should be inferred. We thank the Toronto Community Health Profiles Partnership for providing access to the Ontario Marginalization Index.

## Supporting information


Table S1.
Table S2.
Table S3.
Table S4.
Table S5.
Table S6.

## Data Availability

The datasets from this study are held securely in coded form at ICES. While legal data sharing agreements between ICES and data providers (e.g., healthcare organizations and government) prohibit ICES from making the dataset publicly available, access may be granted to those who meet pre‐specified criteria for confidential access, available at www.ices.on.ca/DAS (email: das@ices.on.ca). The full dataset creation plan and underlying analytic code are available from the authors upon request, understanding that the computer programs may rely upon coding templates or macros that are unique to ICES and are, therefore, either inaccessible or may require modification.
