# Supplementary material for: Income differences in time to colon cancer diagnosis
Source: Cancer Med. 2024 Aug 3;13(15):e6999. doi: 10.1002/cam4.6999 (PMC11297540; doi:10.1002/cam4.6999)
Supplement: Supplementary file 1 — Table S1. Table S2. Table S3. Table S4. Table S5. Table S6. [file CAM4-13-e6999-s001.docx]

Laura E Davis, Erin C Strumpf, Sunil Patel, Alyson L Mahar

**Income inequalities in time to colon cancer diagnosis**

**Supplemental Tables**

**Supplemental Table 1.** Data sources

**Supplemental Table 2.** Covariate definitions

**Supplemental Table 3.** Quantile regression for the effect of neighbourhood income quintile on the diagnostic interval at the 10^th^ percentile

**Supplemental Table 4.** Description of the first encounter for patients with symptomatic diagnostic pathways

**Supplemental Table 5.** Median and 90^th^ percentile diagnostic interval by patient and disease characteristics for asymptomatic pathways

**Supplemental Table 6.** Quantile regression for the effect of neighbourhood income quintile on the diagnostic interval stratified by symptom status and stage at diagnosis (reference for income = Q5)

**Supplemental Table 1. Data sources**

| **Database** | **Description** |
| --- | --- |
| Ontario Cancer Registry (OCR) | The OCR is a passive provincial registry that captures over 95% of incident cancer diagnoses in Ontario since 1964. It includes information on primary cancer site, diagnosis dates, histology, and stage at diagnosis. We used the OCR to capture incident colon cancer diagnoses, date of diagnosis and stage at diagnosis. |
| Ontario Health Insurance Plan Database (OHIP) | OHIP contains billing claims made by all Ontario physicians, including inpatient and outpatient settings. Each claim includes the date, one fee code representing the billable service and one diagnosis code, physician specialty and referring physician where applicable. Physicians are required to submit a diagnosis with each fee code. |
| CIHI Discharge Abstract Database (DAD) and Same Day Surgery Database (SDS) | CIHI DAD and SDS are mandatory reporting systems that provide information on hospital admissions and same-day surgeries. Each record includes up to 20 intervention codes and 25 diagnosis codes. |
| CIHI National Ambulatory Care Reporting System (NACRS) | All emergency department visits in Ontario are captured in NACRS, including administrative, demographic, and clinical data. Each record includes up to 10 intervention codes and 10 diagnosis codes. |
| Registered Persons Database (RPDB) | The RPDB is an ICES database derived from all administrative data sources and provides basic demographic information, such as age, sex, postal code, date of last contact with the healthcare system and OHIP eligibility. |

**Supplemental Table 2. Covariate definitions**

| **Variable** | **Source** | **Definition** | **Type** |
| --- | --- | --- | --- |
| **Socio-demographic characteristics** | | |  |
| Age | RPDB | Age at diagnosis | Categorical: ≤50, 51-60, 61-70, 71-80, >80.  Continuous. |
| Sex | RPDB | Sex | Categorical: Male/Female |
| Rural residence | RPDB | Rural residence was measured by linking postal codes at the time of diagnosis to the Rurality Index of Ontario (RIO). RIO is a function of a region’s population size as well as access to healthcare, such as distance to family practitioners and travel time. | Categorical: Rural (≥45)/Urban (<45) |
| Comorbidities | DAD, SDS, OHIP | Measured using the Elixhauser Comorbidity, which measures hospitalizations in the two years prior to cancer diagnosis. | Categorical: <4; =>4 |
| **Disease characteristics** | | | |
| Histology | OCR | Histology was broadly categorized as adenocarcinoma or non-adenocarcinoma. Adenocarcinoma was defined using the following morphology codes:   - 80003, 81403, 81406, 81443, 81483, 81563, 82013, 82103, 82113, 82133, 82403, 82413, 82433, 82443, 82453, 82463, 82493, 82503, 82533, 82553, 82603, 82613, 82623, 82633, 82653, 83103, 83123, 83233, 83373, 83413, 83803, 84803, 84813, 84903, 85743 | Adenocarcinoma/non-adenocarcinoma |
| Stage at diagnosis | OCR | Stage at diagnosis represents best UICC/AJCC stage which is a combination of Collaborative Staging approach and data from medical records at regional cancer centres. | Categorical: I/II/III/IV/unknown or missing |
| Diagnosis year | OCR | Year of index colon cancer diagnosis. | Categorical: 2007-2019 |
| **Diagnostic interval characteristics** | | | |
| Symptom status | OHIP, DAD, SDS, NACRS | Asymptomatic if the patients received a gFOBT or lower GI endoscopy as their first healthcare encounter and there were no encounters in the ED on the first encounter date.   - Lower GI endoscopy: OHIP = 547, 548, 546, Z555, Z498, Z495, Z494, Z496, Z499, Z497, Z492, Z580, Z571, Z765, Z491, Z570, Z543, Z536, Z535. CIHI = 2NM70^^, 1NM87BA, 1NQ87BA^, 1NM87DA, 1NQ87DA, 2NQ70^^, 2NM70BABG, 2NM70BABH - gFOBT: OHIP = L181, L179, Q152, Q150, G004, Q005 | Categorical: Symptomatic/Asymptomatic |
| ED at index | OHIP, NACRS | First encounter occurs in the ED and that record is present in NACRS or is with an emergency department physician.   - ED physician visit: OHIP = H055, A888, H102, H103, H101, H104, H132, H131, H133, H134, H122, H121, H123, H124, H152, H151, H153, H154, H065, H105, C933, A933 | Categorical: ED/non-ED |
| Lower GI scope | OHIP, DAD, SDS, NACRS | Measured at any time during the diagnostic interval (time from first healthcare encounter to diagnosis)   - Lower GI endoscopy: OHIP = 547, 548, 546, Z555, Z498, Z495, Z494, Z496, Z499, Z497, Z492, Z580, Z571, Z765, Z491, Z570, Z543, Z536, Z535. CIHI = 2NM70^^, 1NM87BA, 1NQ87BA^, 1NM87DA, 1NQ87DA, 2NQ70^^, 2NM70BABG, 2NM70BABH | Categorical: 0/1+ |
| Imaging | OHIP, DAD, SDS, NACRS | Measured at any time during the diagnostic interval.  Colon-cancer related imaging if in any of the following:   - Abdominal or pelvis CT: OHIP = X410, X126, X409, X234, X233, X232, X231, X125, X407, X406. CIHI = 3NM20, 3OT20, 3GY20, 3ZZ20 - Abdominal or pelvis MRI: OHIP = X451, X455, X461, X465. CIHI = 3OT40 - Abdominal or pelvis ultrasound: OHIP = J435, J428, J162, J462, J138, J463, J163, J438, J135, J128. CIHI = 3OT30^^ - Abdominal or pelvis ultrasound: OHIP = X100, X101, X090, X092, X091, X039, X197, X195, X113, X112, X104, X103, X111, X036, X038, X037. CIHI = 3NL10, 3OT10, 3GY10, 3SL10, 3OT12, 3NQ10, 3NZ10, 3NM10, 3NA10, 3NK10, 3SQ10, 3VA10 | Categorical: 0/1+ |
| Diagnostic pathway summary |  | Summary variable using a combination of the following variables defined above: Symptom status, ED at index, lower GI scope and imagining. | Categorical:  1 = Asymptomatic;  2 = Lower GI scope + no imagining + ED at index;  3 = Lower GI scope + no imagining + no ED at index;  4 = Lower GI scope + imagining + ED at index;  5 = Lower GI scope + imagining + no ED at index;  6 = No lower GI scope + imagining + ED at index;  7 = No lower GI scope + imagining + no ED at index;  8 = No lower GI scope + no imagining + ED at index;  9 = No lower GI scope + no imagining + no ED at index; |

**Supplemental Table 3.** Quantile regression for the effect of neighbourhood income quintile on the diagnostic interval at the 10^th^ percentile (Reference = Quintile 5, highest)

|  | **Unadjusted**  **10^th^ percentile** |  | **Adjusted 10^th^ percentile** |  |
| --- | --- | --- | --- | --- |
| **Model** | **Estimate (95% CI)** | **p-value** | **Estimate (95% CI)** | **p-value** |
| **Asymptomatic** | | | | |
| Intercept | 12.00 (9.45-14.55) | <.0001 | 8.11 (2.91-13.32) | 0.0022 |
| Quintile 1 (lowest) | 2.00 (-1.39-5.39) | 0.0266 | 0.56 (-2.32-3.44) | 0.0042 |
| Quintile 2 | 3.00 (-0.61-6.61) |  | 1.82 (-0.97-4.61) |  |
| Quintile 3 | 4.00 (1.08-6.92) |  | 3.88 (1.45-6.31) |  |
| Quintile 4 | 0 (0-0) |  | -0.95 (-3.75-1.85) |  |
| **Symptomatic** | | | | |
| Intercept | 2.00 (2.00-2.00) | <.0001 | 1.51 (0.90-2.13) | <.0001 |
| Quintile 1 (lowest) | 0 (0-0) | 1.0000 | -0.05 (-0.33-0.23) | 0.0958 |
| Quintile 2 | 0 (0-0) |  | 0.30 (-0.07-0.68) |  |
| Quintile 3 | 0 (0-0) |  | 0.32 (-0.07-0.71) |  |
| Quintile 4 | 0 (0-0) |  | 0.29 (-0.09-0.67) |  |

*Abbreviations: CI = confidence interval

*Adjusted models for age, sex rural residence and diagnosis year

*Intercept interpreted as the diagnostic interval at baseline for those at median age (65 years), of male sex, living in urban areas and diagnosed in 2019).

**Supplemental Table 4.** Description of the first encounter for patients with symptomatic diagnostic pathways by neighbourhood income quintile (proportion of total quintile for each category, patients can have multiple first encounters)

| **Encounter Category** | **SYMPTOMATIC (ALL)** | | | | | | | | | |
| --- | --- | --- | --- | --- | --- | --- | --- | --- | --- | --- |
|  | **Q1 (N=10,509)** | | **Q2 (N=10,496)** | | **Q3 (N=9,808)** | | **Q4 (N=9,483)** | | **Q5 (N=9,471)** | |
|  | **N** | **%** | **N** | **%** | **N** | **%** | **N** | **%** | **N** | **%** |
| **Colorectal cancer** | 360 | 3.43 | 315 | 3 | 312 | 3.18 | 313 | 3.3 | 352 | 3.72 |
| **Other cancer** | 344 | 3.27 | 367 | 3.5 | 338 | 3.45 | 339 | 3.57 | 334 | 3.53 |
| **Signs and symptoms Gi** | 4720 | 44.91 | 4775 | 45.49 | 4314 | 43.98 | 4323 | 45.59 | 4381 | 46.26 |
| **Signs and symptoms liver** | 23 | 0.22 | 46 | 0.44 | 30 | 0.31 | 39 | 0.41 | 38 | 0.4 |
| **Signs and symptoms haematology** | 478 | 4.55 | 528 | 5.03 | 458 | 4.67 | 444 | 4.68 | 431 | 4.55 |
| **Anemia** | 1417 | 13.48 | 1390 | 13.24 | 1326 | 13.52 | 1222 | 12.89 | 1109 | 11.71 |
| **Signs and symptoms nutritional** | 42 | 0.4 | 33 | 0.31 | 34 | 0.35 | 47 | 0.5 | 42 | 0.44 |
| **Signs and symptoms bacterial or viral** | 44 | 0.42 | 49 | 0.47 | 30 | 0.31 | 40 | 0.42 | 27 | 0.29 |
| **Abdominal or pelvis MRI** | 20 | 0.19 | 14 | 0.13 | 15 | 0.15 | 18 | 0.19 | 20 | 0.21 |
| **Biopsy** | 773 | 7.36 | 735 | 7 | 729 | 7.43 | 673 | 7.1 | 744 | 7.86 |
| **Abdominal ultrasound** | 215 | 2.05 | 237 | 2.26 | 213 | 2.17 | 214 | 2.26 | 215 | 2.27 |
| **Head or spine MRI** | 7 | 0.07 | 17 | 0.16 | 11 | 0.11 | 5 | 0.05 | 5 | 0.05 |
| **Emergency FP visit** | 2187 | 20.81 | 2115 | 20.15 | 1905 | 19.42 | 1886 | 19.89 | 1700 | 17.95 |
| **Critical care** | 437 | 4.16 | 378 | 3.6 | 343 | 3.5 | 302 | 3.18 | 299 | 3.16 |
| **Gastroenterologist consult** | 182 | 1.73 | 196 | 1.87 | 221 | 2.25 | 194 | 2.05 | 237 | 2.5 |
| **Lower GI scope** | 731 | 6.96 | 729 | 6.95 | 706 | 7.2 | 666 | 7.02 | 757 | 7.99 |
| **Upper GI scope** | 574 | 5.46 | 582 | 5.54 | 515 | 5.25 | 514 | 5.42 | 612 | 6.46 |
| **Non-GI scope** | 24 | 0.23 | 32 | 0.3 | 18 | 0.18 | 18 | 0.19 | 14 | 0.15 |
| **gFOBT** | 14 | 0.13 | 19 | 0.18 | 12 | 0.12 | 10 | 0.11 | 13 | 0.14 |
| **Colon resection** | 464 | 4.42 | 429 | 4.09 | 418 | 4.26 | 395 | 4.17 | 399 | 4.21 |
| **Abdominal xray** | 1528 | 14.54 | 1386 | 13.21 | 1313 | 13.39 | 1242 | 13.1 | 1143 | 12.07 |
| **Other abdominal procedure** | 140 | 1.33 | 131 | 1.25 | 124 | 1.26 | 108 | 1.14 | 109 | 1.15 |
| **Miscellaneous procedure** | 7 | 0.07 | 15 | 0.14 | 10 | 0.1 | 13 | 0.14 | 9 | 0.1 |
| **Abdominal CT** | 1754 | 16.69 | 1698 | 16.18 | 1565 | 15.96 | 1491 | 15.72 | 1420 | 14.99 |
| **Head or spine CT** | 362 | 3.44 | 365 | 3.48 | 328 | 3.34 | 308 | 3.25 | 285 | 3.01 |
| **General surgery consult** | 689 | 6.56 | 636 | 6.06 | 630 | 6.42 | 691 | 7.29 | 681 | 7.19 |
| **Cardiovascular visit** | 543 | 5.17 | 503 | 4.79 | 442 | 4.51 | 438 | 4.62 | 510 | 5.38 |
| **Other consultation** | 181 | 1.6 | 225 | 1.95 | 160 | 1.51 | 190 | 1.9 | 168 | 1.64 |

**Supplemental Table 5. Median and 90^th^ percentile diagnostic interval by symptom status and patient and disease characteristics (asymptomatic pathways only)**

|  | **ASYMPTOMATIC** | | | | | | | | | |
| --- | --- | --- | --- | --- | --- | --- | --- | --- | --- | --- |
|  | **Q1** | | **Q2** | | **Q3** | | **Q4** | | **Q5** | |
| **Variable** | **Median (IQR)** | **90th pct** | **Median (IQR)** | **90th pct** | **Median (IQR)** | **90th pct** | **Median (IQR)** | **90th pct** | **Median (IQR)** | **90th pct** |
| **Diagnostic interval overall** | 71 (36-130) | 222 | 74 (35-137) | 232 | 73 (37-138) | 232 | 68 (33-136) | 232 | 70 (33-144) | 228 |
| **Stage at diagnosis** |  |  |  |  |  |  |  |  |  |  |
| Stage I | 85 (41-141) | 235 | 88 (41-142) | 218 | 81 (44-148) | 223 | 78 (35-141) | 240 | 78 (36-148) | 218 |
| Stage II | 69 (37-130) | 218 | 66 (33-130) | 230 | 65 (36-129) | 222 | 66 (35-135) | 220 | 68 (35-143) | 231 |
| Stage III | 73 (37-128) | 240 | 77 (36-142) | 242 | 67 (35-135) | 232 | 68 (37-129) | 240 | 64 (31-132) | 229 |
| Stage IV | 74 (30-148) | 232 | 65 (30-145) | 253 | 85 (37-159) | 275 | 65 (31-143) | 259 | 85 (35-169) | 255 |
| Stage unknown/missing | 58 (29-113) | 173 | 64 (31-125) | 229 | 70 (35-126) | 202 | 52 (22-127) | 207 | 51 (28-1289) | 205 |
| **Age at index (categorical)** |  |  |  |  |  |  |  |  |  |  |
| <=50 | 70 (29-120) | 194 | 53 (20-115) | 172 | 53 (22-107) | 179 | 44 (18-85) | 167 | 51 (16-123) | 178 |
| 51-60 | 71 (34-125) | 219 | 67 (34-136) | 222 | 67 (33-135) | 219 | 71 (31-140) | 230 | 71 (31-134) | 231 |
| 61-70 | 70 (38-125) | 210 | 72 (33-133) | 220 | 72 (39-131) | 227 | 69 (34-128) | 215 | 70 (33-142) | 219 |
| 71-80 | 70 (33-137) | 239 | 78 (37-140) | 245 | 76 (38-148) | 236 | 67 (35-148) | 255 | 72 (36-150) | 245 |
| >80 | 79 (39-149) | 241 | 80 (39-151) | 250 | 88 (44-164) | 254 | 73 (40-143) | 251 | 69 (37-159) | 235 |
| **Sex** |  |  |  |  |  |  |  |  |  |  |
| Female | 75 (36-141) | 218 | 72 (36-144) | 242 | 72 (39-138) | 245 | 66 (30-137) | 230 | 73 (33-148) | 234 |
| Male | 69 (36-125) | 224 | 76 (34-133) | 224 | 74 (36-138) | 220 | 70 (36-135) | 233 | 65 (33-140) | 225 |
| **RIO at index** |  |  |  |  |  |  |  |  |  |  |
| Urban <45 | 71 (36-132) | 219 | 75 (36-139) | 227 | 73 (37-137) | 232 | 68 (34-136) | 231 | 70 (33-146) | 231 |
| Rural =>45 | 77 (43-125) | 239 | 59 (24-124) | 271 | 67 (39-149) | 228 | 75 (33-141) | 234 | 64 (30-110) | 204 |
| **Elixhauser** |  |  |  |  |  |  |  |  |  |  |
| <4 | 71 (36-128) | 215 | 72 (34-133) | 226 | 72 (36-136) | 227 | 67 (33-133) | 226 | 68 (32-141) | 226 |
| =>4 | 93 (53-172) | 288 | 121 (70-210) | 294 | 91 (44-176) | 268 | 137 (57-196) | 276 | 123 (64-209) | 268 |
| **Histology** |  |  |  |  |  |  |  |  |  |  |
| Other | 85 (36-120) | 244 | 58 (24-125) | 188 | 98 (49-213) | 324 | 44 (17-92) | 164 | 49 (32-149) | 234 |
| Adenocarcinoma | 71 (36-131) | 222 | 74 (35-137) | 233 | 72 (37-137) | 227 | 69 (34-137) | 232 | 70 (33-144) | 228 |
| **Received at least one lower GI scope** |  |  |  |  |  |  |  |  |  |  |
| Tom0 | 86 (47-157) | 276 | 82 (38-148) | 250 | 89 (46-166) | 278 | 78 (36-160) | 264 | 75 (37-156) | 255 |
| 1+ | 70 (35-127) | 212 | 72 (34-133) | 225 | 71 (36-133) | 217 | 66 (33-133) | 222 | 68 (32-142) | 224 |

**Supplemental Table 6. Quantile regression for the effect of neighbourhood income quintile on the diagnostic interval. Stratified by symptom status and stage at diagnosis.** Multivariable intercept represents the estimated diagnostic interval in days at baseline (for an individual with mean age 71, male, income quintile 5, stage unknown, diagnosed in 2019)

|  | **UNADJUSTED** | | | | **ADJUSTED** | | | |
| --- | --- | --- | --- | --- | --- | --- | --- | --- |
|  | **50^th^ percentile** |  | **90^th^ percentile** |  | **50^th^ percentile** |  | **90^th^ percentile** |  |
| **Model** | **Estimate (95% CI)** | **Wald p-value** | **Estimate (95% CI)** | **Wald p-value** | **Estimate (95% CI)** | **Wald p-value** | **Estimate (95% CI)** | **Wald p-value** |
| **ASYMPTOMATIC** | | | | | | | | |
| **Stage 1** |  |  |  |  |  |  |  |  |
| **Intercept** | 78.00 (68.49-87.15) | <.0001 | 217.69 (201.25-234.13) | <.0001 | 54.78 (27.37-82.19) | <.0001 | 121.75 (62.34-181.15) | <.0001 |
| **Q1** | 7.00 (-6.37-20.37) | 0.4027 | 17.31 (-17.59-52.20) | 0.6643 | 7.32 (-3.75-18.40) | 0.1938 | -8.50 (-25.94-8.94) | 0.5398 |
| **Q2** | 10.00 (-2.29-22.29) |  | 0.31 (-26.96-27.58) |  | 10.11 (0.23-19.98) |  | -8.25 (-23.74-7.24) |  |
| **Q3** | 3.00 (-8.04-14.04) |  | 5.31 (-19.63-30.25) |  | 10.11 (-0.18-20.40) |  | -3.00 (-18.91-12.91) |  |
| **Q4** | 0.00 (-13.84-13.84) |  | 22.31 (-10.89-55.51) |  | 2.04 (-8.87-12.95) |  | 4.25 (-13.33-21.83) |  |
| **Stage 2** |  |  |  |  |  |  |  |  |
| **Intercept** | 68.00 (61.02-74.98) | <.0001 | 231.00 (200.07-261.93) | <.0001 | 39.00 (18.98-59.01) | 0.0001 | 134.16 (101.86-166.46) | <.0001 |
| **Q1** | 1.00 (-9.21-11.21) | 0.8806 | -13.00 (-59.97-33.97) | 0.9624 | 4.09 (-5.20-13.38) | 0.9102 | -7.18 (-22.97-8.61) | 0.3869 |
| **Q2** | -2.00 (-11.46-7.46) |  | -1.00 (-64.86-62.86) |  | 0.92 (-8.64-10.48) |  | -1.42 (-16.44-13.59) |  |
| **Q3** | -3.00 (-11.39-5.39) |  | -9.00 (-49.59-31.59) |  | 3.73 (-6.35-13.81) |  | -15.39 (-33.14-2.35) |  |
| **Q4** | -2.00 (-11.17-7.17) |  | -11.00 (-50.17-28.17) |  | 2.09 (-7.72-11.90) |  | -8.00 (-24.37-8.37) |  |
| **Stage 3** |  |  |  |  |  |  |  |  |
| **Intercept** | 64.00 (57.02-70.98) | <.0001 | 229.00 (201.56-256.44) | <.0001 | 44.81 (21.10-68.52) | 0.0002 | 167.32 (131.22-203.42) | <.0001 |
| **Q1** | 9.26 (-0.62-19.14) | 0.0238 | 10.12 (-30.08-50.32) | 0.9556 | 10.25 (0.49-20.00) | 0.1299 | -2.33 (-21.69-17.03) | 0.8655 |
| **Q2** | 13.08 (4.37-21.78) |  | 13.00 (-24.41-50.41) |  | 12.00 (0.56-23.44) |  | 7.53 (-9.90-24.96) |  |
| **Q3** | 3.00 (-7.62-13.62) |  | 3.00 (-29.25-35.25) |  | 5.43 (-4.21-15.08) |  | 3.08 (-17.10-23.26) |  |
| **Q4** | 4.00 (-5.56-13.56) |  | 11.00 (-32.14-54.14) |  | 4.43 (-5.42-14.29) |  | 1.79 (-16.58-20.16) |  |
| **Stage 4** |  |  |  |  |  |  |  |  |
| **Intercept** | 85.00 (70.53-99.47) | <.0001 | 255.00 (224.12-285.88) | <.0001 | 108.91 (67.63-150.18) | <.0001 | 176.77 (127.88-225.65) | <.0001 |
| **Q1** | -12.29 (-31.33-6.74) | 0.0290 | -23.00 (-72.15-26.15) | 0.5181 | -11.91 (32.47-8.65) | 0.1876 | -21.47 (-42.33-(-0.60)) | 0.0896 |
| **Q2** | -20.00 (-41.02-1.02) |  | -2.00 (-58.96-54.96) |  | -15.62 (-31.59-0.35 |  | -16.23 (-37.29-4.83) |  |
| **Q3** | 0.00 (-20.72-20.72) |  | 20.00 (-25.45-65.45) |  | -8.26 (-27.54-11.03) |  | 4.60 (-17.41-26.62) |  |
| **Q4** | -19.58 (-36.19-(-2.96)) |  | 4.00 (-36.92-44.92) |  | -18.57 (-35.15-(-1.99) |  | -10.88 (-30.13-8.36) |  |
| **Stage unknown** |  |  |  |  |  |  |  |  |
| **Intercept** | 51.32 (43.15-59.49) | <.0001 | 205.00 (163.76-246.24) | <.0001 | 45.59 (29.74-61.44) | <.0001 | 131.57 (99.61-163.53) | <.0001 |
| **Q1** | 6.68 (-2.78-16.14) | 0.0130 | -32.00 (-87.37-23.37) | 0.1581 | 4.64 (-6.31-15.59) | 0.0117 | -26.00 (-47.22-(-4.78) | 0.0277 |
| **Q2** | 12.68 (0.60-24.76) |  | 24.00 (-23.71-71.71) |  | 8.68 (-2.25-19.61) |  | -11.32 (-35.36-12.73) |  |
| **Q3** | 18.68 (6.33-31.03) |  | -3.00 (-64.42-58.42) |  | 15.44 (3.58-27.30) |  | 4.95 (-20.45-30.35) |  |
| **Q4** | 0.68 (-14.43-15.80) |  | 2.00 (-55.09-59.09) |  | -4.20 (-15.71-7.31) |  | -11.47 (-36.84-13.89) |  |
| **SYMPTOMATIC** | | | | | | | | |
| **Stage 1** |  |  |  |  |  |  |  |  |
| **Intercept** | 153.00 (145.46-160.54) | <.0001 | 400.00 (387.80-412.20) | <.0001 | 113.52 (71.55-155.48) | <.0001 | 435.94 (374.47-497.41) | <.0001 |
| **Q1** | 7.00 (-3.39-17.39) | 0.3191 | 8.00 (-8.57-24.57) | 0.5740 | 6.44 (-6.42-19.30) | 0.6874 | 5.60 (-9.66-20.86) | 0.7480 |
| **Q2** | 0.00 (-11.37-11.37) |  | 7.00 (-8.74-22.74) |  | 0.86 (-11.86-13.58) |  | 6.78 (-7.30-20.85) |  |
| **Q3** | -5.00 (-15.90-5.90) |  | -4.00 (-23.22-15.22) |  | -3.79 (-16.47-8.90) |  | 4.71 (-9.86-19.29) |  |
| **Q4** | 0.17 (-12.48-12.83) |  | 5.00 (-12.53-22.53) |  | -0.15 (-13.02-12.71) |  | 10.31 (-4.76-25.38) |  |
| **Stage 2** |  |  |  |  |  |  |  |  |
| **Intercept** | 116.00 (108.86-123.14) | <.0001 | 388.00 (373.95-402.05) | <.0001 | 98.09 (69.18-127.01) | <.0001 | 395.89 (357.45-434.33) | <.0001 |
| **Q1** | 5.00 (-4.99-14.99) | 0.1636 | 24.00 (5.76-42.24) | 0.0089 | 5.85 (-6.54-18.25) | 0.3688 | 14.67 (0.47-28.88) | 0.2024 |
| **Q2** | 9.00 (-0.29-18.29) |  | 25.00 (6.90-43.10) |  | 5.47 (-5.02-15.96) |  | 7.06 (6.72-20.83) |  |
| **Q3** | 8.00 (-3.05-19.05) |  | 16.00 (-2.05-34.05) |  | 8.46 (-2.09-19.00) |  | 5.60 (-10.16-21.37) |  |
| **Q4** | -1.00 (-11.33-9.33) |  | 4.00 (-14.19-22.19) |  | -0.95 (-11.71-9.81) |  | -1.28 (-15.79-13.22) |  |
| **Stage 3** |  |  |  |  |  |  |  |  |
| **Intercept** | 113.00 (105.26-120.74) | <.0001 | 383.00 (371.80-394.20) | <.0001 | 114.29 (93.62-134.96) | <.0001 | 423.14 (385.04-461.25) | <.0001 |
| **Q1** | 13.00 (1.43-24.57) | 0.1417 | 22.00 (6.11-37.89) | 0.0206 | 11.78 (2.46-21.09) | 0.0552 | 23.09 (7.74-38.44) | 0.0515 |
| **Q2** | 6.00 (-5.75-17.75) |  | 17.00 (2.28-31.73) |  | 2.83 (-8.92-14.58) |  | 12.65 (-3.51-28.80) |  |
| **Q3** | 3.00 (-6.84-12.84) |  | 24.00 (7.02-40.98) |  | 1.78 (-8.70-12.25) |  | 16.41 (0.40-32.42) |  |
| **Q4** | 0.00 (-9.79-9.79) |  | 19.00 (2.37-35.63) |  | -1.62 (-13.00-9.76) |  | 18.32 (2.32-34.32) |  |
| **Stage 4** |  |  |  |  |  |  |  |  |
| **Intercept** | 59.00 (49.07-68.93) | <.0001 | 360.00 (340.20-379.80) | <.0001 | 62.25 (40.42-84.08) | <.0001 | 373.61 (332.53-414.69) | <.0001 |
| **Q1** | 26.00 (8.02-43.98) | 0.0164 | 22.00 (-2.76-46.76) | 0.1724 | 14.70 (2.91-26.50) | 0.0020 | 10.60 (-11.65-32.85) | 0.5007 |
| **Q2** | 23.00 (8.24-37.76) |  | 25.00 (2.12-47.88) |  | 18.91 (9.26-28.57) |  | 15.36 (-5.92-36.64) |  |
| **Q3** | 20.00 (5.53-34.47) |  | 28.00 (3.16-52.84) |  | 16.50 (4.56-28.44) |  | 15.24 (-2.25-32.73) |  |
| **Q4** | 11.00 (-2.29-24.29) |  | 16.00 (-9.39-41.39) |  | 7.86 (-3.99-19.70) |  | 8.04 (-12.60-28.68) |  |
| **Stage unknown** |  |  |  |  |  |  |  |  |
| **Intercept** | 137.00 (126.08-147.92) | <.0001 | 428.00 (413.84-442.16) | <.0001 | 104.02 (83.60-124.45) | <.0001 | 387.05 (356.17-417.93) | <.0001 |
| **Q1** | 8.00 (-9.88-25.88) | 0.9239 | 7.00 (-14.73-28.73) | 0.6671 | 6.85 (-11.91-25.61) | 0.8381 | 10.92 (-17.67-39.51) | 0.3793 |
| **Q2** | 1.00 (-13.60-15.60) |  | 6.00 (-20.42-32.42) |  | -1.34 (-17.12-14.44) |  | 18.92 (-8.30-46.13) |  |
| **Q3** | 2.39 (-14.14-18.91) |  | 6.00 (-12.65-24.65) |  | 2.13 (-16.65-20.92) |  | 4.00 (-19.00-27.00) |  |
| **Q4** | 3.00 (-11.56-17.56) |  | -5.00 (-24.83-14.83) |  | 5.19 (-12.13-22.51) |  | -7.00 (-35.49-21.49) |  |
